# Supplementary material for: Conditional chemoconnectomics (cCCTomics) as a strategy for efficient and conditional targeting of chemical transmission
Source: eLife. 2024 Apr 30;12:RP91927. doi: 10.7554/eLife.91927 (PMC11060718; doi:10.7554/eLife.91927)
Supplement: Figure 3—source data 3. [file elife-91927-fig3-data3.zip › Figure 3 source data3 part 2.zip!/Figure 3-Source data 3-part2/1025-13-CRISPResso_Report_ack040l3.zip!/Nucleotide_percentage_quilt_around_sgRNA_AGCACAAACTTGGCGGTGAC.pdf]

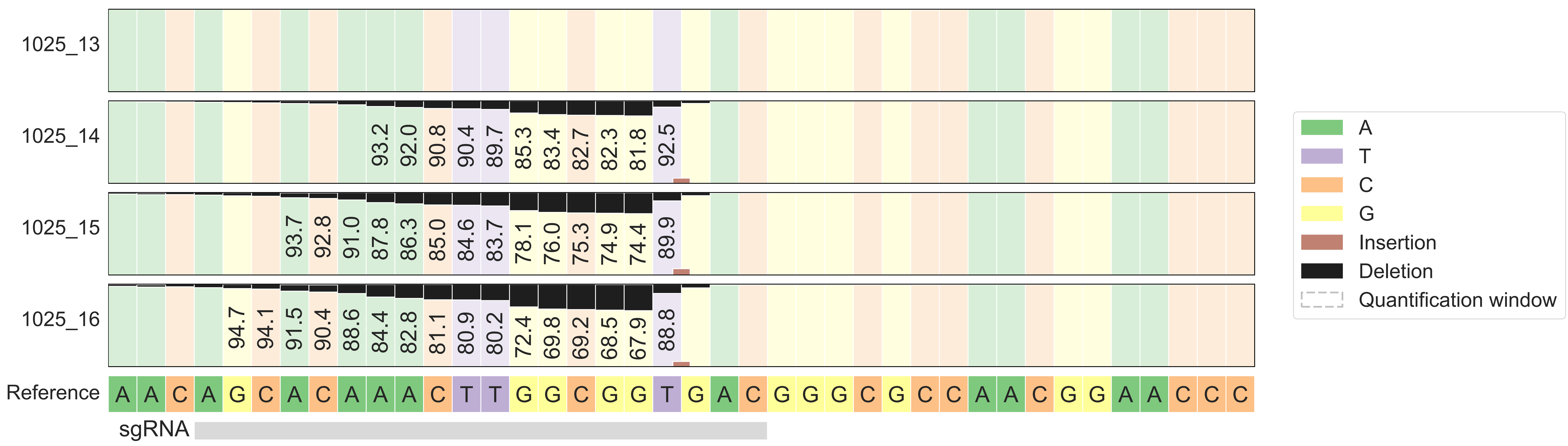
1025_13
1025_14
93.2 92.0 90.8 90.4 89.7 85.3 83.4 82.7 82.3 81.8 92.5
1025_15
93.7 92.8 91.0 87.8 86.3 85.0 84.6 83.7 78.1 76.0 75.3 74.9 74.4 89.9
1025_16
94.7 94.1 91.5 90.4 88.6 84.4 82.8 81.1 80.9 80.2 72.4 69.8 69.2 68.5 67.9 88.8
Reference
A A C A G C A C A A A C T T G G C G G T G A C G G G C G C C A A C G G A A C C C
sgRNA
A
T
C
G
Insertion
Deletion
Quantification window
